# Field evaluation of sexed and conventional semen in Holstein–Friesian cows: Linking semen quality with fertility and calf sex ratio across climatic zones

**DOI:** 10.14202/vetworld.2026.1085-1096

**Published:** 2026-03-17

**Authors:** Putri Utami, Aulia Puspita Anugra Yekti, Habib Asshidiq Syah, Anggita Dian Pramudhita, Tri Agus Siswoyo, Nurul Isnaini, Trinil Susilawati

**Affiliations:** 1 Doctoral Student, Department of Animal Reproduction and Breeding, Faculty of Animal Science, Universitas Brawijaya, Malang, Indonesia; 2 Department of Animal Reproduction and Breeding, Faculty of Animal Science, Universitas Brawijaya, Malang, Indonesia; 3 Postgraduate Student, Department of Animal Reproduction and Breeding, Faculty of Animal Science, Universitas Brawijaya, Malang, Indonesia; 4 Graduate Program of Biotechnology, the Center of Excellence on Crop Industrial Biotechnology (PUI-PT BioTIn), Universitas Jember, Jember, Indonesia

**Keywords:** artificial insemination, calf sex ratio, dairy cattle fertility, Holstein–Friesian cows, reproductive performance, semen quality, sexed semen, smallholder dairy systems

## Abstract

**Background and Aim:**

Sexed semen is increasingly used in dairy breeding programs to enhance herd productivity by increasing the proportion of female calves. However, concerns remain regarding possible trade-offs between semen processing, post-thaw quality, fertility outcomes, and calf sex ratio under field conditions, particularly in smallholder production systems and varying climates. This study evaluated laboratory semen quality and field reproductive performance of sexed and conventional semen in Holstein–Friesian cows across different climatic zones.

**Materials and Methods:**

The study combined laboratory semen evaluation and a multisite field trial. Semen was processed using Percoll density gradient centrifugation (PGDC) for sexing and subsequently cryopreserved. Post-thaw quality parameters, including motility, viability, abnormalities, concentration, plasma membrane integrity, intact acrosome cap, total motile spermatozoa, and DNA fragmentation, were assessed and compared with conventional semen using an independent t-test. Field performance was evaluated in 300 clinically healthy cows from three smallholder dairy locations representing low, moderate, and high temperature–humidity index zones. Cows were randomly assigned to receive single- or double dose artificial insemination with either semen type. Non-return rate, conception rate, calving rate, and calf sex ratio were analyzed using Fisher’s exact and chi-square tests, with significance set at p < 0.05.

**Results:**

Sexed semen showed motility comparable to conventional semen, while viability, concentration, plasma membrane integrity, and intact acrosome cap differed significantly between treatments (p < 0.05). Abnormalities, total motile spermatozoa, and DNA fragmentation were not significantly different (p > 0.05). Across locations, non-return rate, conception rate, and calving rate did not differ significantly between semen types or dosing strategies (p > 0.05). Nevertheless, reproductive performance tended to be higher in the cooler climatic zones. Sexed semen produced a higher proportion of female calves than conventional semen, indicating successful enrichment of X-bearing spermatozoa under field conditions.

**Conclusion:**

Sexed semen produced through PGDC maintains acceptable post-thaw quality and field fertility comparable to conventional semen while improving the likelihood of female calf production. Climatic conditions, particularly lower temperature–humidity index, appear to support better reproductive outcomes. These findings support the practical use of sexed semen in smallholder dairy systems, although long-term multilocation studies integrating environmental and management factors are recommended to optimize reproductive efficiency and sustainability.

## INTRODUCTION

Artificial insemination (AI) remains a vital tool in advancing dairy cattle reproduction, being widely used to enhance genetic merit, optimize herd structure, and improve productivity [1]. The use of sexed semen, which enables producers to predetermine the sex of offspring, typically favoring heifer calves to support milk production systems, is a significant development within this technology [2]. Flow cytometry is the most widely commercialized among various technologies used for semen sexing [3]. However, Percoll density gradient centrifugation (PGDC) has emerged as an alternative technique for spermatozoa sex selection in research and specific field applications [4]. This method separates spermatozoa cells based on their density differences, offering a cost-effective and simple option for field use [5]. Despite its practicality, the use of Percoll-based sexed semen raises critical questions regarding its post-thaw quality, fertilizing potential, and consistency under field conditions. In general, sexed semen exhibits reduced spermatozoa concentration, motility, viability, and increased susceptibility to environmental stressors; all of these factors may compromise AI success.

In many developing countries, including Indonesia, the majority of beef and dairy cattle production originates from smallholder or community-based farming systems rather than large commercial farms. These smallholder farms form the backbone of livestock production and significantly contribute to food security, rural livelihoods, and the local economy. However, reproductive efficiency in such settings is often constrained by limited access to advanced reproductive technologies, suboptimal management, and environmental stressors [6]. Consequently, optimizing AI protocols and technologies, such as sexed semen, within the context of smallholder farms is critical for enhancing productivity and sustainability.

Although sexed semen technology has been widely investigated, most previous studies have focused primarily on laboratory-based semen evaluation or controlled experimental herds, with limited integration of semen processing quality, field fertility outcomes, and calf sex ratio within real smallholder production systems. In particular, evidence linking post-thaw semen quality parameters to reproductive performance under diverse climatic conditions remains insufficient. Smallholder dairy farms, which dominate cattle production in many developing countries, often operate under variable environmental stress, nutritional limitations, and manage-ment constraints that may influence AI success differently from commercial farms. Furthermore, alternative semen sexing approaches such as PGDC have received comparatively less field validation than flow cytometry–based systems, especially regarding their ability to maintain semen functional quality while achieving desirable fertility and sex ratio outcomes. Consequently, there remains a need for comprehensive multisite studies that simultaneously evaluate laboratory semen quality, field reproductive performance, and calf sex ratio to better understand the practical reliability of sexed semen technologies under smallholder and climate-variable dairy production conditions.

Therefore, this study aimed to comprehensively evaluate the effectiveness of sexed semen produced through PGDC compared with conventional semen in Holstein–Friesian (HF) cows across different climatic zones. Specifically, the study sought to (i) compare post-thaw semen quality parameters, including motility, viability, abnormalities, concentration, plasma membrane integrity, intact acrosome cap, total motile spermatozoa, and DNA fragmentation; (ii) assess field reproductive performance based on non-return rate, conception rate, and calving rate under smallholder farm conditions; and (iii) determine the resulting calf sex ratio to evaluate the practical success of sex selection. By integrating laboratory assessments with multisite field outcomes, this study aimed to clarify the relationship between semen functional quality and reproductive efficiency and to provide evidence-based guidance for the application of sexed semen technology in climate-diverse smallholder dairy systems.

## MATERIALS AND METHODS

### Ethical approval

All experimental procedures involving animals were reviewed and approved by the Animal Care and Use Committee of Universitas Brawijaya, Malang, Indonesia, under ethical clearance number 18-KEP-UB-2024.The study was conducted in accordance with the institutional guidelines for the care and use of animals in research and adhered to national regulations governing livestock experimentation in Indonesia.

Semen collection procedures were performed at the Singosari National Artificial Insemination Center (SNAIC), Malang, Indonesia, an ISO 9001:2015-certified institution, following standard operational protocols for bull handling, semen collection, processing, cryopreservation, and quality control. All procedures involving bulls were conducted by trained personnel to minimize stress and ensure animal welfare. Artificial vagina collection was performed under hygienic conditions without causing pain or injury to the animals.

Field AI procedures were carried out by licensed and trained inseminators using the conventional transrectal technique in clinically healthy HF cows. Only non-pregnant cows with normal reproductive tracts and body condition scores between 3 and 4 were included in the study. Estrus detection was performed through behavioral observation and veterinary examination to ensure appropriate timing of insemination and avoid unnecessary interventions.

Vitamin supplementation (Rheinvit AD_3_E Plus; Rheinvet, Düsseldorf, Germany) was administered intramuscularly as part of routine reproductive management to support animal health and did not exceed recommended dosages. No experimental surgical procedures, hormonal synchronization beyond routine farm practice, or invasive interventions were performed.

All animals were managed under existing smallholder farming systems, and no cow was subjected to procedures outside standard dairy reproductive management practices. Animal handling during rectal palpation for pregnancy diagnosis (60 days post-AI) was performed by licensed veterinarians to minimize discomfort and stress. Any cow exhibiting signs of illness or reproductive complications was referred for appropriate veterinary care and excluded from further experimental evaluation if necessary.

Written informed consent was obtained from all participating farmers prior to animal enrollment. Farmers were informed about the study objectives, procedures, potential benefits, and possible risks. Participation was voluntary, and farmers retained the right to withdraw their animals from the study at any time without penalty.

The study design ensured that animal welfare, biosecurity measures, and ethical research standards were maintained throughout laboratory and field components.

### Study period and location

Field data collection was conducted from September 2023 to September 2024. The study included assessments of post-thaw semen quality, conception rate, and calf sex ratio. The study was conducted at three smallholder dairy farming areas in Malang Regency, East Java, Indonesia, Ngantang, Bantur, and Jabung, which served as field-based sites for AI implementation. Climatic conditions were characterized using the temperature–humidity index (THI) [7]. Climatic classification was based on daily ambient temperature and relative humidity recorded during the study period and used to calculate THI for each location. Based on mean THI values, Ngantang was classified as a low THI zone (THI = 68–71), Jabung as a moderate THI zone (THI = 72–77), and Bantur as a high THI zone (THI = 78–80). All farms operated under smallholder systems, with farmers managing 2–5 HF cows and practicing routine AI under forage-based feeding systems. Laboratory assessments were conducted at SNAIC Malang, Indonesia, and at the Animal Reproduction Laboratory, Faculty of Animal Science, Universitas Brawijaya, Malang, Indonesia.

### Animals

The study involved 300 clinically healthy, non-pregnant HF cows selected from smallholder dairy farms across three sub-districts, with 100 cows enrolled per location. Cows had parity values ranging from 1 to 5 and were managed under similar small-scale production systems. Inclusion criteria included a normal reproductive tract, clear estrus expression before AI, and a body condition score (BCS) between 3 and 4 on a 1–5 scale. BCS was assessed using standard dairy cattle scoring methods through visual evaluation and palpation of fat reserves over the ribs, loin, and tailhead [8]. Non-pregnant status and estrus confirmation were verified using farm records, behavioral observations (mounting, standing estrus, mucus discharge, reddening of the vulva) [9], and rectal examination by licensed veterinarians. Following selection, cows were randomly assigned to receive AI using either sexed or conventional semen.

### Experimental design

This study consisted of two components: laboratory testing of semen quality and a field experiment at the smallholder farm level. In the laboratory, semen quality was assessed for two treatments (n = 10): conventional semen (T1) and sexed semen containing X chromosome–bearing spermatozoa (T2). Parameters analyzed included motility, viability, abnormalities, concentration, total motile spermatozoa, plasma membrane integrity, and DNA fragmentation (evaluated using the toluidine blue method). The laboratory study evaluated the effects of cryopreservation and sexing technology on semen quality.

In the field experiment, AI was performed on 300 HF cows distributed across three locations (100 cows per location). At each location, cows were randomly divided into four treatment groups:G0: conventional semen, single dose (25 cows);G1: conventional semen, double dose (25 cows);G2: sexed semen, single dose (25 cows);G3: sexed semen, double dose (25 cows).

Pregnancy success was evaluated using non-return rate 1 (NRR-1), non-return rate 2 (NRR-2), conception rate (CR), calving rate (CvR), and sex ratio (SxR) of the newborn calf [10].

### Semen collection, processing, freezing, and thawing

Semen was obtained from two HF bulls, GW Amish (2 years old, 698 kg) and Mate (2 years old, 730 kg). Semen collection was performed twice weekly using an artificial vagina (AV; IMV Technologies, L’Aigle, France). The AV was filled with warm water (450–500 mL) at 40°C–42°C. The inner part of the AV was lubricated with sterile lubricating gel from the outer opening to one-third of the upper part, ensuring the outer part was not touched directly to maintain hygienic conditions.

Fresh semen was immediately evaluated macroscopically and microscopically. Only semen with individual motility ≥70% and abnormalities <20% was used for further processing. Semen was processed using the PGDC method. The sexing procedure involved layering ten Percoll medium gradients (Sigma-Aldrich, St. Louis, MO, USA) in a centrifuge tube (Pyrex, Jakarta, Indonesia), each containing 0.5 mL with concentrations ranging from 20% to 60%, arranged from lowest to highest concentration.

**Figure 1:**
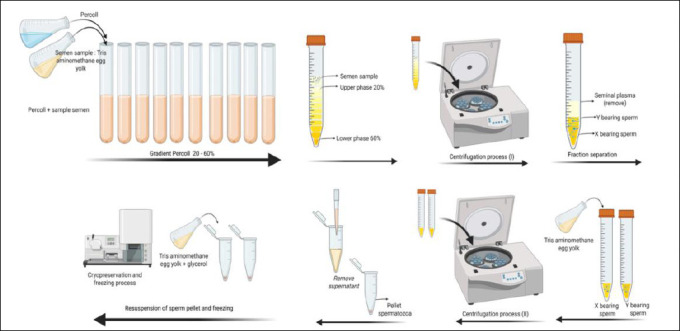
Schematic overview of the Percoll density gradient centrifugation procedure used for semen.

Fresh semen samples (2 mL; 1.653 × 10^9^ spermatozoa) were placed above the gradient and centrifuged (Hettich, Tuttlingen, Germany) at 291 × *g* for 7 min. Fractions were then combined with 3 mL of Tris-amino-methane diluent containing egg yolk and centrifuged again at 148.1 × *g* for 5 min. The supernatant was discarded, and the pellet was resuspended in egg yolk Tris-aminomethane buffer supplemented with glycerol for cryopreservation. The final suspension was packaged into 0.25 mL plastic straws (~25 × 10^6^ spermatozoa), sealed, and gradually cooled to 5°C for 4 h (IMV Technologies). After the cooling process, the straws were frozen using the nitrogen vapor method for 10 min at a distance of 4 cm from the liquid nitrogen surface and then placed in a liquid nitrogen container (−196°C) for long-term storage (Figure 1). Before AI and quality testing, frozen semen was thawed by immersion in water at 37°C for 30 s.

### Spermatozoa analysis

Individual motility was assessed based on progressive movement in five microscopic fields at 400× magnification (Olympus CX-23, Tokyo, Japan). One drop of semen was placed on an object glass (One Lab, Shanghai, China) and incubated on a warming plate (IKA, Staufen, Germany) at 37°C [11].

Viability was evaluated using eosin–nigrosin staining, counting 200 spermatozoa across ten random fields [12]. Semen concentration was determined using a hemocytometer after dilution (1:100) in 3% NaCl within a Neubauer chamber (Marienfeld, Lauda-Königshofen, Germany). Abnormal morphology was assessed using eosin–nigrosin staining based on 200 observed cells. Total motile spermatozoa were calculated from concentration and motility percentage.

The hypoosmotic swelling test (HOST) was used to evaluate plasma membrane integrity [13]. Twenty microliters of semen were incubated in hypoosmotic solution (1:10) for 30 min at 37°C and observed microscopically at 400× (Olympus BX-33, Tokyo, Japan). Curled tails indicated intact membranes. Intact acrosome cap was evaluated using a 1% formalin–NaCl mixture, followed by staining and observation of 200 cells.

DNA fragmentation was assessed using toluidine blue staining. Samples were washed with phosphate-buffered saline with Tween, fixed with ethanol–acetone, hydrolyzed with 0.1 N HCl, stained with 0.05% toluidine blue solution, dehydrated, and examined at 400× (Olympus BX-33). Bright blue staining indicated intact DNA, whereas dark blue staining indicated DNA damage.

### AI

Cows showing estrus were administered 20 mL Rheinvit AD_3_E Plus vitamin (Rheinvet, Düsseldorf, Germany) intramuscularly per animal. This supplementation prevented deficiencies of vitamins A, D_3_, and E and supported reproductive and immune function. Some cows had previously recovered from foot and mouth disease (FMD) during the 2022 outbreak.

Frozen semen was thawed at 37°C for 30 s. AI was performed either as a single or double dose. In the single dose protocol, one straw was administered at the first sign of estrus. In the double dose protocol, cows received one straw at estrus onset and another 8 h later. AI was performed using the conventional transrectal method, and semen was deposited in the uterine corpus according to established international protocols [14, 15].

### Reproductive performance

Reproductive performance was evaluated using NRR1, NRR2, CR, CvR, and SxR. CR was calculated as the proportion of cows diagnosed pregnant at first insemination multiplied by 100%. Pregnancy diagnosis was performed by rectal palpation 60 days post-AI. Non-return rate was calculated based on cows not showing repeat estrus during days 19–21 (NRR1) and 38–40 (NRR2) post-AI [15]. Calving rate represented the proportion of live-born calves relative to inseminated cows [16]. Sex ratio was determined from the proportion of male and female calves born.

### Statistical analysis

Spermatozoa quality data were analyzed using the Shapiro–Wilk normality test, Levene’s homogeneity test, and independent t-test. Reproductive performance data were analyzed using Fisher’s exact test for individual locations and Pearson’s chi-square test for pooled analysis. All analyses were performed using R software version 4.3.3, with significance set at α = 0.05.

## RESULTS

### Sperm quality

Statistical analysis using the independent t-test showed significant differences between conventional semen and sexed semen for several sperm quality parameters (p < 0.05). Sperm viability, concentration, plasma membrane integrity, and intact acrosome capacity significantly differed among treatments. In contrast, MOT, TMS, sperm abnormalities, and DNA fragmentation did not significantly differ between the groups (p > 0.05) (Table 1).

**Table 1:** Quality parameters of Holstein–Friesian bull spermatozoa in conventional and sexed semen.

Parameter	T1 (Mean ± SD)	T2 (Mean ± SD)	p-value	Interpretation
MOT (%)	40.80 ± 2.57	41.30 ± 2.11	0.6405	Non-significant
Viability (%)	65.08 ± 2.94	67.95 ± 2.31	0.0257*	Significant
Abnormality (%)	8.02 ± 2.65	8.01 ± 1.65	0.9881	Non-significant
Concentration (×10^6^/mL)	26.78 ± 2.26	24.53 ± 1.44	0.0163*	Significant
TMS (×10^6^/mL)	10.96 ± 1.47	10.14 ± 0.93	0.1549	Non-significant
PMI (%)	65.89 ± 2.68	68.75 ± 1.97	0.0139*	Significant
IAC (%)	65.21 ± 2.55	68.18 ± 2.57	0.0184*	Significant
DNA fragmentation (%)	11.50 ± 1.20	10.60 ± 1.49	0.1509	Non-significant

T1: Conventional semen, T2: Sexed semen, SD: Standard deviation, TMS: Total motile spermatozoa, PMI: Plasma membrane integrity, IAC: Intact acrosome cap. p < 0.05 indicates significance (*).

### Reproductive performance

Statistical analysis using Fisher’s exact test for each location and Pearson’s chi-square test for the pooled data showed no significant differences among treatments with respect to NRR1, NRR2, CR, and CvR (p > 0.05) (Table 2). This pattern was consistent across all three study locations. Although no statistically significant differences were detected, a higher pregnancy outcome was observed at the Ngantang site, which showed the highest CR and CvR among the locations (Figures 3 and 4). Sexed semen resulted in a higher proportion of female calves than conventional semen, although differences were not significant in some treatments (p > 0.05).

**Table 2:** Reproductive performance of Holstein–Friesian cows by location and artificial insemination treatment.

Parameter	Location	G0 (%)	G1 (%)	G2 (%)	G3 (%)	p-value
NRR1	Ngantang	68 (17/25)	92 (23/25)	64 (16/25)	76 (19/25)	0.092
	Jabung	76 (19/25)	60 (15/25)	68 (17/25)	76 (19/25)	0.596
	Bantur	68 (17/25)	72 (18/25)	64 (16/25)	60 (15/25)	0.893
NRR2	Ngantang	64 (16/25)	80 (20/25)	56 (14/25)	60 (15/25)	0.318
	Jabung	48 (12/25)	40 (10/25)	36 (9/25)	48 (12/25)	0.806
	Bantur	48 (12/25)	56 (14/25)	52 (13/25)	44 (11/25)	0.907
CR	Ngantang	40 (10/25)	48 (12/25)	20 (5/25)	44 (11/25)	0.168
	Jabung	24 (6/25)	24 (6/25)	28 (7/25)	32 (8/25)	0.959
	Bantur	24 (6/25)	32 (8/25)	36 (9/25)	32 (8/25)	0.871
CvR	Ngantang	36 (9/25)	48 (12/25)	20 (5/25)	44 (11/25)	0.189
	Jabung	24 (6/25)	24 (6/25)	28 (5/25)	32 (8/25)	0.959
	Bantur	24 (6/25)	24 (6/25)	32 (8/25)	20 (5/25)	0.959

Values are shown as percentages followed by the number of successes in parentheses. NRR1: Non-return rate 1, NRR2: Non-return rate 2, CR: Conception rate, CvR: Calving rate, G0: Conventional semen single dose, G1: Conventional semen double dose, G2: Sexed semen single dose, G3: Sexed semen double dose.

**Figure 2:**
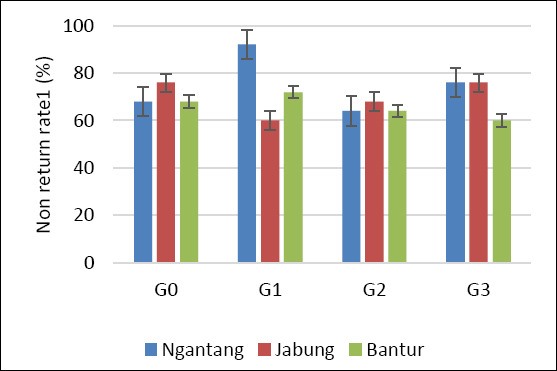
Non-return rate 1 of Holstein–Friesian cows across four AI treatments and locations. G0: Conventional semen, single dose; G1: Conventional semen, double dose; G2: Sexed semen, single dose; G3: Sexed semen, double dose. AI: Artificial insemination.

**Figure 3:**
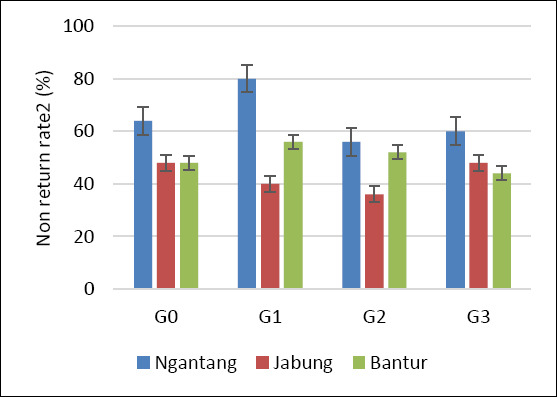
Non-return rate 2 of Holstein–Friesian cows across four AI treatments and locations. G0: Conventional semen, single dose; G1: Conventional semen, double dose; G2: Sexed semen, single dose; G3: Sexed semen, double dose. AI: Artificial insemination.

**Figure 4:**
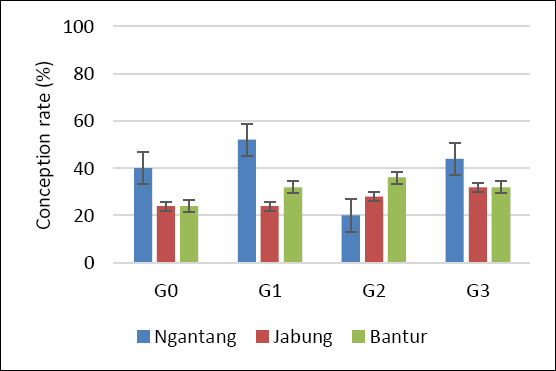
Conception rate of Holstein–Friesian cows across four AI treatments and locations. G0: Conventional semen, single dose; G1: Conventional semen, double dose; G2: Sexed semen, single dose; G3: Sexed semen, double dose. AI: Artificial insemination.

## DISCUSSION

### Post-thaw motility and viability of sexed semen

Spermatozoa motility is a key parameter for assessing the ability of spermatozoa to move actively and progressively toward the oocyte for fertilization [17]. In the present study, the average motility of spermatozoa in T1 and T2 did not differ significantly, and both values exceeded the minimum recommended standard for frozen semen (approximately 40%) [18], indicating suitability for AI. Despite the mechanical stress associated with Percoll centrifugation, motility remained adequate to support fertility, consistent with reports indicating that optimized Percoll centrifugation protocols can preserve spermatozoa motility [19]. In contrast, the viability of spermatozoa was higher in sexed semen than in conventional semen, which may be explained by the density gradient sorting mechanism enriching fractions with more intact plasma membranes and higher metabolic activity [20]. This finding aligns with evidence that density gradient methods can act as a natural selection process by removing dead or damaged spermatozoa during separation [21]. PGDC is a separation technique based on density differences among components in a spermatozoa suspension, with high-quality spermatozoa (motile with good membrane integrity) differing in density from damaged or non-motile cells. During cryopreservation, spermatozoa experience oxidative stress and mechanical injury due to ice crystal formation, which can compromise the plasma membrane and organelles such as mitochondria [22].

### Morphology and sperm concentration after PDGC

The percentage of abnormal spermatozoa did not differ between sexed and conventional semen, with both treatments showing similar abnormality values (approximately 8%). This suggests that processing using the PDGC method does not adversely affect spermatozoa morphology [23]. Density-based separation does not appear to induce major deformation of the head, midpiece, or tail structures, thereby maintaining morphological quality suitable for AI. Conversely, spermatozoa concentration was significantly lower in sexed semen than in conventional semen, which is consistent with the principle of density gradient centrifugation whereby only spermatozoa with superior motility and morphology traverse the gradient, resulting in reduced recovery of total cells [24]. Density-based separation selectively enriches viable spermatozoa while reducing the overall number of recovered cells, consistent with earlier findings [25]. In line with this mechanism, total sperm motility showed a decreasing trend in sexed semen compared with conventional semen; however, the values remained within acceptable limits for AI. The reduction in sperm yield following PDGC is likely attributable to stringent sperm selection and unavoidable losses during washing and gradient processing [26].

### Plasma membrane integrity and intact acrosome cap

Plasma membrane integrity reflects the ability of spermatozoa to maintain physiological function during fertilization. The present findings indicate that PGDC does not substantially compromise plasma membrane integrity. Preserved membrane function is essential for ion regulation, zona pellucida recognition, and membrane fusion during fertilization [27]. Damage to the plasma membrane has been associated with reduced progressive motility and decreased fertility following AI [28]. In addition, intact acrosome caps indicate readiness for the acrosome reaction, which releases hydrolytic enzymes required for zona pellucida penetration [29]. The observed preservation of acrosome integrity suggests that density-based separation supports acrosomal structure maintenance, consistent with previous reports. In AI programs, an intact acrosome is critical for successful oocyte penetration and fertilization [30, 31].

### DNA fragmentation and genetic integrity

DNA fragmentation is widely recognized as an important sperm quality indicator because of its association with embryo development and pregnancy outcomes following AI [32]. In this study, both sexed and conventional semen exhibited low DNA fragmentation, indicating good preservation of genetic integrity. DNA damage in spermatozoa may arise from oxidative stress, cryopreservation-induced chromatin alterations, and suboptimal post-thaw handling [33, 34]. The present findings support the concept that density-based separation can aid selection of spermatozoa with compact chromatin and intact DNA while minimizing mechanical and oxidative injury. This is relevant because elevated DNA fragmentation has been linked to compromised embryo quality and impaired blastocyst development, even when fertilization can still occur [35].

### Field fertility outcomes across climatic zones

Reproductive performance in cows is influenced by multiple interacting factors, including biological characteristics (breed, age, and reproductive status) [36], management practices (estrus detection or hormonal protocols) [37], semen type (fresh, chilled, or frozen) [38], insemination timing relative to estrus, semen dose, deposition site (corpus uteri vs. cornua uteri), and inseminator skill and experience [39]. In the present study, overall reproductive performance (NRR1, NRR2, and CR) across treatments and locations was generally below 60% (Figures 2, 3, and 4). Among the three locations, Ngantang (low THI) exhibited relatively higher CR than Jabung and Bantur, and G3 in Ngantang showed the highest success rate, although still below 60%. These findings suggest that more favorable thermal conditions may support improved fertility outcomes in smallholder dairy systems.

### Role of THI and heat stress in reproductive efficiency

The highest CR was observed in Ngantang, exceeding that in Jabung and Bantur, likely due to differences in geography and climate. Ngantang, a highland area with cooler temperatures and more stable humidity (THI = 68–71), provides physiologically favorable conditions for cows by supporting thermoregulation and reducing heat stress, thereby helping maintain reproductive function [7]. In contrast, Jabung and Bantur are lowland areas with higher temperatures and greater humidity fluctuations, contributing to elevated THI and heat stress. Heat stress disrupts endocrine regulation, including reduced luteinizing hormone and estradiol secretion, which are critical for ovulation, and may compromise follicular development and oocyte quality [40].

### Pregnancy losses and smallholder field constraints

**Figure 5:**
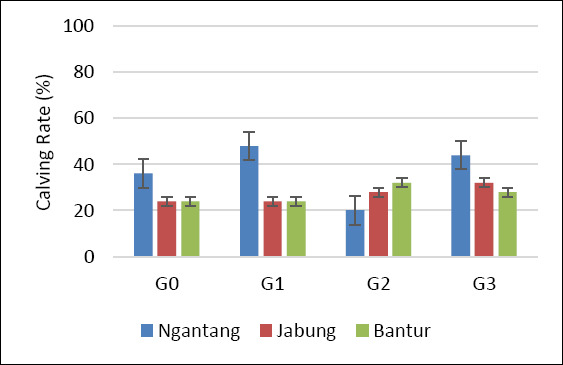
Calving rate of Holstein–Friesian cows across four AI treatments and locations. G0: Conventional semen, single dose; G1: Conventional semen, double dose; G2: Sexed semen, single dose; G3: Sexed semen, double dose. AI: Artificial insemination.

**Figure 6:**
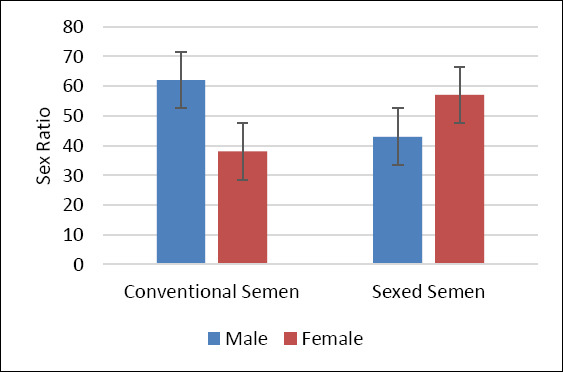
Sex ratio of calves born from different semen types in Holstein–Friesian dairy cows.

Birth records indicated that not all confirmed pregnancies resulted in live calf delivery (Figures 5 and 6). In the sexed semen group, the proportion of female calves was higher, accounting for 57%, while male calves accounted for 43%. In contrast, calves produced from conventional semen showed a higher proportion of males (62%) compared with females (38%). Pregnancy losses in this study were mainly associated with abortion and maternal mortality, both of which reduce reproductive efficiency in dairy cows [50–52]. Abortion may be linked to infectious reproductive diseases, endocrine disruption, nutritional deficiencies, and environmental stressors that impair pregnancy maintenance [41]. Maternal mortality in late gestation or around parturition may be associated with metabolic disorders, dystocia, or inadequate management of systemic conditions [42]. Notably, some cows had a history of FMD during the national outbreak in 2022 and were clinically recovered and eligible at the time of AI. However, prior exposure to FMD may contribute to residual impacts on physiological condition, feed intake behavior, and BCS, potentially increasing the risk of abortion or maternal mortality and reducing pregnancy maintenance [43]. These observations highlight that, under smallholder conditions, reproductive evaluation should consider conception outcomes together with pregnancy maintenance and live calf delivery as integrated indicators of reproductive efficiency.

### Calf sex ratio and effectiveness of PDGC sexing

Calf sex outcomes showed a descriptive shift in sex ratio between conventional and sexed semen. Conventional semen tended to yield a higher proportion of male calves, whereas sexed semen increased the proportion of female calves, suggesting partial enrichment of X-bearing spermatozoa using PGDC. However, the achieved proportion did not reach the expected threshold typically reported for advanced sexing technologies. The Percoll-based mechanism relies on differences in DNA content between X- and Y-bearing spermatozoa, with X-bearing spermatozoa having slightly higher density due to greater DNA content [43]. Density gradient layers enable partial stratification of spermatozoa populations into fractions enriched for the targeted sex chromosome [3, 4]. Similar trends have been described in previous density-based sexing studies, although accuracy generally remains lower than that achieved using flow cytometry–based sexing [43–45]. The moderate effectiveness observed may be influenced by biological and technical factors, including repeated centrifugation steps that can reduce motility and viability through mechanical stress and medium exposure, potentially affecting fertilization capacity of X-bearing spermatozoa [46].

### Timing of insemination and semen deposition considerations

Fertilization outcomes are strongly influenced by insemination timing relative to ovulation because X- and Y-bearing spermatozoa may differ in motility and lifespan in the female reproductive tract. Conditions within the tract, including cervical mucus pH, hormonal status, and reproductive condition, also affect spermatozoa survival and transport [47]. These factors are closely linked to herd management, including nutrition, environmental conditions, and reproductive health programs. Inseminator skill remains a critical determinant of AI success, particularly when sexed semen is used [48]. In this study, semen was deposited in the uterine corpus using the four-deposition technique to support distribution to both uterine horns. Although deep horn deposition has been suggested to improve fertilization efficiency in some settings, corpus deposition was applied here to maintain balanced distribution and acceptable fertilization potential when using sexed semen [49, 50].

## CONCLUSION

The present study demonstrated that sexed semen produced using PGDC maintains post-thaw functional quality comparable to conventional semen in HF cows. Although significant differences were observed in sperm viability, concentration, plasma membrane integrity, and intact acrosome cap, motility, abnormalities, total motile spermatozoa, and DNA fragmentation did not differ significantly between treatments. Under field conditions across three climatic zones, reproductive performance indicators, including non-return rate, CR, and CvR, were broadly similar between sexed and conventional semen, indicating that the use of sexed semen does not compromise fertility outcomes. Importantly, sexed semen increased the proportion of female calves, confirming its effectiveness for targeted herd replacement strategies. Fertility outcomes tended to be higher in the cooler highland environment, highlighting the influence of temperature–humidity index and environmental management on reproductive success.

From a practical perspective, these findings support the application of sexed semen technology in smallholder dairy systems, where improving the proportion of female calves can enhance herd productivity, genetic progress, and long-term farm sustainability. The results indicate that PDGC-based sexed semen offers a relatively simple and cost-effective alternative to advanced sorting technologies while maintaining acceptable fertility under field conditions.

A major strength of this study lies in its integrated design, combining laboratory semen quality evaluation with multisite field fertility assessment across distinct climatic conditions. This approach provides a realistic evaluation of technology performance in smallholder production systems. However, several limitations should be acknowledged. The study involved a limited number of bulls and locations, and management practices, health history (including prior FMD exposure), and nutritional variability among farms may have influenced reproductive outcomes. In addition, long-term reproductive performance, calf growth, and economic efficiency were not assessed.

Future research should include larger multisite trials involving multiple bulls, extended monitoring across successive breeding cycles, and integration of nutritional, environmental, and management interventions to optimize reproductive efficiency. Comparative evaluation with flow cytometry–sorted semen and economic cost–benefit analyses would also strengthen practical recommendations.

In conclusion, Percoll-based sexed semen represents a reliable reproductive technology for dairy cattle breeding under smallholder conditions, capable of maintaining fertility while improving the likelihood of female calf production. Its adoption, combined with improved herd management and climate-adaptive practices, may contribute substantially to sustainable dairy development.

## DATA AVAILABILITY

All the generated data are included in the manuscript.

## AUTHORS’ CONTRIBUTIONS

PU: Conceptualized and designed the study, collected and analyzed the data, drafted and revised the manuscript. APAY: Conceptualized and designed the study and reviewed the manuscript. HAS and ADP: Analyzed the data and drafted the manuscript. TAS, NI, and TS: Supervised and designed the study and reviewed the manuscript. All authors have read and approved the final version of the manuscript.
